# Fear conditioning and stimulus generalization in association with age in children and adolescents

**DOI:** 10.1007/s00787-021-01797-4

**Published:** 2021-05-13

**Authors:** Julia Reinhard, Anna Slyschak, Miriam A. Schiele, Marta Andreatta, Katharina Kneer, Andreas Reif, Katharina Domschke, Matthias Gamer, Paul Pauli, Jürgen Deckert, Marcel Romanos

**Affiliations:** 1 Center of Mental Health, Department of Child and Adolescent Psychiatry, Psychosomatics and Psychotherapy, University Hospital of Würzburg, Würzburg, Germany; 2 Department of Psychiatry and Psychotherapy, Medical Center-University of Freiburg, Faculty of Medicine, University of Freiburg, Freiburg, Germany; 3 Department of Psychology (Biological Psychology, Clinical Psychology and Psychotherapy), University of Würzburg, Würzburg, Germany; 4 Department of Psychology, Education and Child Studies, Erasmus University Rotterdam, Rotterdam, The Netherlands; 5 Department of Psychiatry, Psychosomatic Medicine and Psychotherapy, University Hospital of Frankfurt, Frankfurt, Germany; 6 Department of Psychology (Experimental Clinical Psychology), University of Würzburg, Würzburg, Germany; 7 Center of Mental Health, Department of Psychiatry, Psychosomatics and Psychotherapy, University Hospital of Würzburg, Würzburg, Germany

**Keywords:** Fear conditioning, Fear generalization, Overgeneralization, Anxiety disorders, Childhood and adolescence, Development

## Abstract

The aim of the study was to investigate age-related differences in fear learning and generalization in healthy children and adolescents (*n* = 133), aged 8–17 years, using an aversive discriminative fear conditioning and generalization paradigm adapted from Lau et al. (2008). In the current task, participants underwent 24 trials of discriminative conditioning of two female faces with neutral facial expressions, with (CS+) or without (CS−) a 95-dB loud female scream, presented simultaneously with a fearful facial expression (US). The discriminative conditioning was followed by 72 generalization trials (12 CS+, 12 GS1, 12 GS2, 12 GS3, 12 GS4, and 12 CS−): four generalization stimuli depicting gradual morphs from CS+ to CS− in 20%-steps were created for the generalization phases. We hypothesized that generalization in children and adolescents is negatively correlated with age. The subjective ratings of valence, arousal, and US expectancy (the probability of an aversive noise following each stimulus), as well as skin conductance responses (SCRs) were measured. Repeated-measures ANOVAs on ratings and SCR amplitudes were calculated with the within-subject factors stimulus type (CS+, CS−, GS1-4) and phase (Pre-Acquisition, Acquisition 1, Acquisition 2, Generalization 1, Generalization 2). To analyze the modulatory role of age, we additionally calculated ANCOVAs considering age as covariate. Results indicated that (1) subjective and physiological responses were generally lower with increasing age irrespective to the stimulus quality, and (2) stimulus discrimination improved with increasing age paralleled by reduced overgeneralization in older individuals. Longitudinal follow-up studies are required to analyze fear generalization with regard to brain maturational aspects and clarify whether overgeneralization of conditioned fear promotes the development of anxiety disorders or vice versa.

**Supplementary Information:**

The online version contains supplementary material available at 10.1007/s00787-021-01797-4.

## Introduction

The transition from childhood to adolescence is a high-risk period for the development of anxiety and mood disorders [1, 2]. Estimates of childhood and adolescent anxiety disorders ranged from 5.3 to 17% [3]. Since childhood and adolescent anxiety poses a risk for the development of anxiety disorders in adulthood [4], and since pathological anxiety has high comorbidity rates with other disorders [5], advancing our understanding of the development of fear and the pathogenesis of anxiety disorders is paramount for targeted prevention and therapy strategies.

In the development of fear and the pathogenesis of anxiety disorders, fear conditioning and its generalization are considered to be crucial learning mechanisms [6], and may thus serve as apt translational model mechanisms of clinically relevant fear acquisition [7]. Since Pavlov´s seminal work on classical conditioning, many studies concerning fear conditioning in animals and humans have been published [6, 8–12]. Fear conditioning describes the process by which a particular neutral stimulus, e.g., a neutral face, is associated with an aversive stimulus (the unconditioned stimulus: US), e.g., an electrical shock or an unpleasant sound. Thereby the neutral stimulus becomes a conditioned stimulus (CS) and results in the expression of a fear response (conditioned response: CR) even after stopping the presentation of the aversive stimulus. Furthermore, differential fear conditioning refers to learning that a conditioned stimulus (CS+) predicts an aversive event, while another stimulus (CS−) is never followed by the US and therefore predicts safety.

On a neuronal level, the differential fear learning process entails two different processes: first, brain structures developing early in life and at a phylogenetically early stage, e.g., amygdala and hippocampus, are involved when confronted with a potentially threatening stimulus [13, 14]. Second, cortical brain regions that developed at later evolutionary stages and mature later during ontogenesis (e.g., prefrontal cortex) are crucial to inhibit fear responses to ambiguous safety cues, as this is especially relevant in fear generalization processes [15, 16]. Fear generalization describes the process whereby (conditioned) fear responses extend to stimuli (generalization stimuli: GSs) more or less similar to CS+ yet never associated with the US. The generalization gradient usually changes as a function of reduced similarity between GSs and CS+ [17] resulting in a steep, quadratic versus a shallow, linear gradient indicating limited versus strong generalization, respectively.

Overgeneralization indicated by a linear vs. quadratic gradient is associated with anxiety disorders [18, 19] and anxious personality [20] indicating a plausible role of fear generalization in the pathogenesis of anxiety disorders. However, most studies concerning fear generalization were conducted in adult participants [18, 19, 21]—possibly due to ethical limitations in children in the choice of an effective US—thus limiting our insight into early developmental stages of pathological fear and anxiety. Since anxiety disorders usually develop during childhood [22], it is paramount to investigate fear generalization processes in children with a special focus on developmental aspects.

Only a few studies have been published on fear learning and generalization in children and adolescents so far [10, 23–25]. These studies suggest (a) stronger fear responses in anxious youth compared to non-anxious controls [26] as well as (b) overgeneralization in healthy children compared to adults [23]. As both human and animal studies suggest that developmental progress reduces generalization and sharpens discrimination [23, 27], we assumed that overgeneralization in children was mostly due to maturational aspects concerning the prefrontal cortex. Thus, a maturational lack of the prefrontal inhibitory function on neural circuits may correlate with overgeneralization in children [23]. Specifically, the ventromedial prefrontal cortex (PFC), which is one of the last brain regions to mature [28], has been hypothesized to mediate inhibitory top–down control [16].

Fear per se is a highly adaptive mechanism allowing us to react quickly and appropriately when encountering threat, and generalization of fear to ambiguous stimuli is a protective mechanism promoting cautious behavior in childhood according to the “better safe than sorry” principle [29, 30]. However, overgeneralization of conditioned fear in adulthood has been linked to anxiety disorders [18, 19]. Hence, the persistence of fear overgeneralization from childhood into adulthood may pose a potential risk mechanism contributing to the development of pathological anxiety. However, there is still a substantial knowledge gap concerning the developmental trajectories of fear generalization.

Since childhood is a highly vulnerable period for the development of anxiety disorders [22, 31, 32], the question arose as to whether characteristic fear generalization evolves across childhood and adolescence and if overgeneralization is correlated with age in youth. In a previous study of our research group, we found that children aged 8–10 years showed heightened fear generalization when compared to healthy adults [23]. However, it remains unknown what changes occur at what time within the transition from childhood (from 10 years on) to adulthood. In other words: how do fear generalization gradients change in relation to age? Thus, with the current study, we looked at the association between age and aspects of fear learning, more precisely fear conditioning and fear generalization, within a wider age range from 8 to 17 years.

## Methods

### Participants

A total of *n* = 188 healthy children and adolescents participated in the study and were recruited from primary/secondary schools mostly within the framework of the collaborative research center SFB-TRR-58 subproject Z02 in the Department of Child and Adolescent Psychiatry at the University Hospital of Würzburg, Germany. All participants were native German speakers. Exclusion criteria were a manifest or lifetime DSM-IV axis l disorder (ascertained using the German versions of the Diagnostic Interview for Mental Disorders for Children and Adolescents, Kinder-DIPS, [33]), intake of psychoactive medication, and an IQ < 85 determined by the German version of the Culture Fair Intelligence Test 2 [34]. Nine participants had to be excluded from the analysis, because they withdrew their consent during the experiment, and further 46 participants were excluded due to technical errors and/or low or non-response during the physiological recordings (i.e., response < 0.02 µS). Thus, the final sample consisted of *n* = 133 children and adolescents (70 female; aged 8–17 years; mean age: 12.27 years, SD = 2.82 years; for further descriptive data see Suppl Table 1).

The study was approved by the ethical committee of the Medical Faculty of the Julius-Maximilian-University of Würzburg (study numbers 211/16 and 106/10) and complied with the latest version of the declaration of Helsinki. All participants as well as their parents gave written informed consent and each family was paid € 30 compensation for their participation.

### Task and stimulus material

#### Stimuli (CS+ , CS−, GS1-4)

Stimulus presentation was controlled using Presentation software version 17.2 (Neurobehavioral Systems, Inc., Albany, CA, USA). We used a modified version of the “screaming lady paradigm” by Lau et al. [8] as described in Schiele, Reinhard et al. [17] (see Suppl Fig. 1). In this task, pictures of two actresses with neutral facial expression were presented (NimStim Face Stimulus Set; [35]) that served as either the CS+ or CS−, with one of the two faces being randomly selected as the CS+ for each participant. Four generalization stimuli depicting gradual morphs from CS+ to CS− in 20%-steps (GS1-4) were created using the graphics software Sqirlz Morph Version 2.1 (Xiberpix, Solihull, UK). The unconditioned stimulus (US) was a 95-dB female scream (International Affective Digital Sounds system), presented simultaneously with a fearful facial expression of the same actress assigned as the CS+.

#### Design

The experiment was divided into three consecutive phases: pre-acquisition, acquisition, and generalization separated by ratings. Pre-acquisition consisted of four CS+ and four CS – presentations, while no US appeared. During acquisition, 12 CS+ and 12 CS− were presented. The CS+ was paired with the US on 10 trials. The generalization phase consisted of 12 CS+ , 12 CS−, and 12 of each of the four GSs. Half the CS+ trials were followed by the US to prevent premature extinction. CS− and all GSs were never paired with the US. CSs and GSs were presented for 6 s each. The US was presented immediately following CS+ offset for 1.5 s. Inter-trial intervals (ITI) varied from 9 to 12 s, during which a white fixation cross was displayed centrally on the screen. Acquisition and generalization trials were separated into two phases, each containing half of the trials per phase, that is 6 presentations per stimulus category. Stimulus order was pseudo-randomized, so that the same stimulus could not appear more than twice in a row. Participants were instructed to passively view pictures of two female faces, and that an unpleasant sound would be heard occasionally, but they were not informed of the CS-US contingencies. They were told that it would be possible to become startled and/or frightened and that participation could be discontinued at any time.

#### Ratings

Following pre-acquisition, acquisition, and generalization, participants rated each stimulus on arousal, valence, and US expectancy on the computer screen. Arousal and valence ratings were indicated on 9-point Likert scales, ranging from “very calm” (1) to “very arousing” (9), and “very unpleasant” (1) to “very pleasant” (9), respectively. US expectancy was recorded in percent on a scale from 1 to 100 in 10% increments as the probability of an aversive noise following each stimulus (from “certainly not” (1) to “very certain” (11)).

### Physiological recordings and data reduction

Skin conductance data were recorded continuously throughout the experiment using a constant current of 0.5 V. Electrodes (5 mm Ag/AgCl electrodes) were placed on the thenar and hypothenar eminences of the non-dominant hand [36]. A V-Amp 16 amplifier and Vision Recorder Software were used (BrainProducts Inc., Gilching, Germany). Sampling rate was set to 1000 Hz. The electrodermal data were analyzed offline using Vision Analyzer 2 software (BrainProducts, Gilching, Germany). The signal was first filtered offline with a high cutoff filter of 1 Hz and a notch filter of 50 Hz. Skin conductance responses (SCRs) were then defined as the base-to-peak differences (in µS) between the onset (900–4000 ms after stimulus onset) and the peak (2000–6000 ms after stimulus onset) of the first response [36] and resulting SCRs were checked manually. A minimum response criterion of 0.02 µS was applied, with lower responses scored as 0. SCR data were then normalized following an approach described by Dunsmoor et al. [37], that is, by computing generalization gradients for each phase and block as a function of the response to one stimulus type relative to the sum of responses to all stimuli. That is, for each of the pre-acquisition, acquisition, and generalization phases, the sum of SCRs to each stimulus was divided by the sum of responses to all stimuli, resulting in an index for each stimulus type that allows for the direct comparison of generalization patterns between groups.

### Statistical analysis

All statistical analyses were performed with IBM SPSS (Version 25, SPSS Inc.). To verify learning effects due to conditioning, we first calculated four separated 2 × 3 repeated-measures ANOVAs on ratings and SCR amplitudes, respectively, with the within-subject factors stimulus type (CS+ , CS−) and phase (Pre-acquisition, Acquisition 1, Acquisition 2). To analyze generalization effects, we calculated four separated 6 × 2 repeated-measures ANOVAs for ratings and SCR amplitudes, respectively, again with the within-subject factors stimulus type (CS+ , GS1-4, CS−) and phase (Generalization 1, Generalization 2). To investigate the generalization gradient in more detail, we conducted trend analysis for all four variables (ratings of arousal, valence, US expectancy, and SCR). To analyze the modulatory role of age on anxiety learning and its generalization processes, we than calculated ANCOVAs considering age as covariate. To follow up on significant age effects, we calculated Pearson correlation coefficients (two-tailed). Greenhouse–Geisser corrections for non-sphericity were performed where indicated, though uncorrected degrees of freedom are reported for the sake of better readability. In case of significant interaction effects, post hoc *t*-tests were calculated and all post hoc tests were Bonferroni corrected. Corrected *p* values, and partial *η*^2^ for significant results are reported. Alpha was set at 0.05.

## Results

### Pre-acquisition/acquisition phases

#### Ratings

Main effects of stimulus type and phase as well as significant stimulus type × phase interactions on arousal and US expectancy ratings were found (Suppl Table 2), indicating that the conditioning procedure was successful. Similarly, for valence ratings, a significant main effect of stimulus type and a significant stimulus type × phase interaction were found (Suppl Table 2). Post hoc tests for the significant stimulus type × phase interaction on arousal ratings indicated that there were significant differences between the stimuli (CS+ /CS−) after Acquisition 1 (*t* (132) = 7.11, *p* < .001), and Acquisition 2 (*t* (132) = 7.19, *p* < .001), but not after Pre-acquisition (*t* (132) = 0.43, *p* = .669, Suppl Fig. 2). Similarly, for valence ratings, stimuli (CS+ /CS−) were rated significantly different after Acquisition 1 (*t* (132) = 3.82, *p* < .001), and Acquisition 2 (*t* (132) = 5.79, *p* < .001), but not after Pre-acquisition (*t* (132) = 1.43, *p* = .154). Likewise, US expectancy ratings differed significantly between CS+ and CS− during Acquisition (Acquisition 1: *t* (132) = 1.98, *p* = .05; Acquisition 2: *t* (132) = 9.04, *p* < .001), but not prior to conditioning (*t* (132) = 0.07, *p* = .942).

**Fig. 1:**
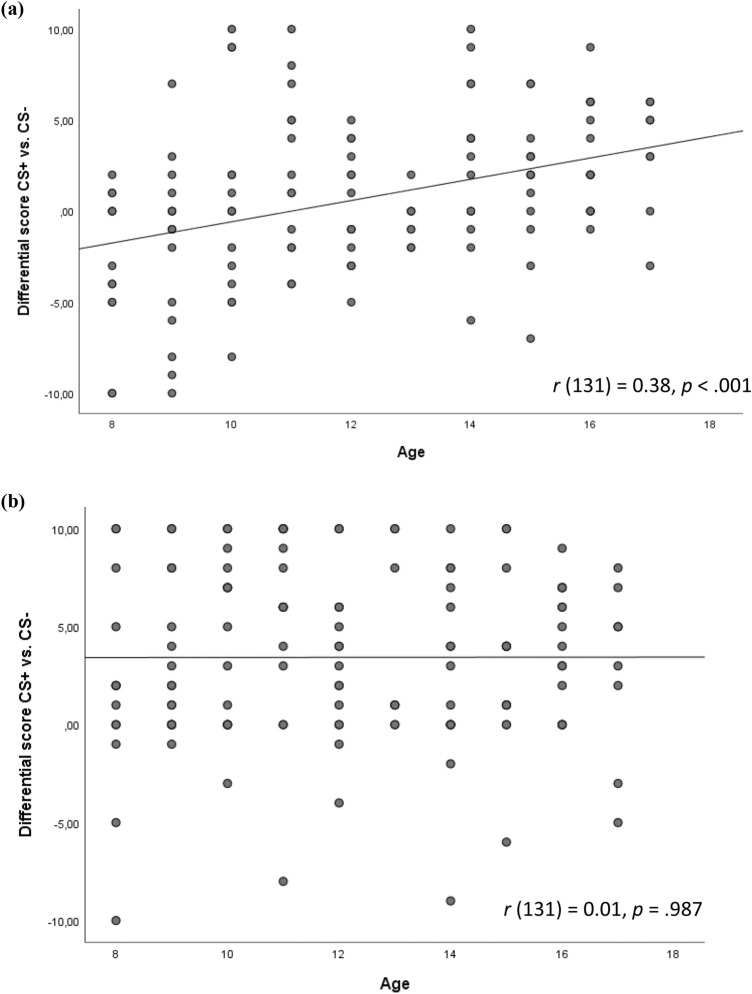
Correlations between age and differential scores between CS+ and CS− in US expectancy ratings in (**a**) Acquisition 1 (ACQ1) and (**b**) Acquisition 2 (ACQ2). The significant positive correlation indicates better discrimination between CS+ and CS− in older participants after ACQ 1. There was no significant correlation with age, however, after ACQ 2

#### Physiological response

Similar to the ratings, for the SCR amplitudes, we found significant main effects of stimulus type and phase, but no significant stimulus type × phase interaction (Suppl Table 2), indicating that participants showed stronger psychophysiological arousal to CS+ vs. CS−, but generally highest SCR after Acquisition 1 and lowest SCR after Acquisition 2, irrespective of the stimulus type.

#### Effects of age

Age significantly modulated arousal ratings, valence ratings, US expectancy ratings, as well as the SCR (Table 1): the main effect of age in the arousal and US expectancy ratings as well as in the SCR was based on a negative correlation (arousal: *r* (131) = − 0.26, *p* = .003; US expectancy: *r* (131) = − 0.21, *p* = .016; SCR: *r* (131) = − 0.28, *p* = .001), meaning that older participants showed lower ratings and SCR.

**Table 1:** Results of ANCOVAs at pre-acquisition/acquisition

	Main effect of age	Stimulus type × age	Phase × age	Stimulus type × phase × age
Arousal	*F* (1,131) = 6.03, *p* = .015, *η*^2^ = .04	*F* (1,131) = 1.48, *p* = .227, *η*^2^ = .01	*F* (2,235) = 5.62, *p* = .006, *η*^2^ = .04	*F* (2,240) = 2.94, *p* = .059, *η*^2^ = .02
Valence	*F* (1,131) = 2.67, *p* = .105, *η*^2^ = .02	*F* (1,131) = 0.47, *p* = .496, *η*^2^ = .004	*F* (2,231) = 0.64, *p* = .508, *η*^2^ = .01	*F* (2,246) = 9.06, *p* < .001, *η*^2^ = .07
US expectancy	*F* (1,131) = 12.70, *p* = .001, *η*^2^ = .09	*F* (1,131) = 5.52, *p* = .020, *η*^2^ = .04	*F* (2,221) = 3.88, *p* = .029, *η*^2^ = .03	*F* (2,262) = 11.90, *p* < .001, *η*^2^ = .08
SCR	*F* (1,131) = 10.48, *p* = .002, *η*^2^ = .07	*F* (1,131) = 0.42, *p* = .516, *η*^2^ = .003	*F* (2,236) = 0.41, *p* = .643, *η*^2^ = .003	*F* (2,238) = 0.27, *p* = .741, *η*^2^ = .002

Results concerning age on arousal, valence and US expectancy ratings as well as on skin conductance response (SCR)

We then calculated correlations with age for those effects involving the factor stimulus type following an approach described by Andreatta et al. [38]. Thus, for the stimulus type × phase × age three-way interaction concerning valence ratings (*F* (2,246) = 9.06, *p* < .001, *η*^2^ = .07, Table 1), we calculated differential scores between CS+ and CS− for Acquisition 1 and Acquisition 2, respectively, and then subtracted the differential score of Acquisition 1 from the differential score of Acquisition 2 (ACQ 2 [CS+ minus CS−] − ACQ 1[CS+ minus CS−]). This differential score was then correlated with age. We found no significant correlation, however (*r* (131) = 0.10, *p* = .277).

For the stimulus type × age two-way interaction concerning US expectancy ratings (*F* (1,131) = 5.52, *p* = .020, *η*^2^ = .04, Table 1), we first calculated the average of Acquisition 1 and Acquisition 2 separately for CS+ and CS−, respectively. Then, we correlated age with the differential score between CS+ and CS−. We found a significant positive correlation (*r* (131) = 0.23, *p* = .009) indicating that the older the participants were, the better they differentiated between the stimuli.

To follow up on the stimulus type × phase × age three-way interaction concerning US expectancy ratings (*F* (2,262) = 11.90, *p* < .001, *η*^2^ = .08), we again calculated differential scores between CS+ and CS− for Acquisition 1 and Acquisition 2 respectively, and then subtracted the differential score of Acquisition 1 from the differential score of Acquisition 2 (ACQ 2 [CS+ minus CS−] − ACQ 1[CS+ minus CS−]). We found a significant negative correlation (*r* (131) = − 0.34, *p* < .001) between this differential score and age indicating that the older the participants were, the lower the discriminative US expectancy ratings were indicated after Acquisition 2 as compared to Acquisition 1.

To disentangle the correlational effects, we first correlated age with the difference scores between CS+ and CS− separately after Acquisition 1 and Acquisition 2, and second, we correlated the difference scores between Acquisition 1 and Acquisition 2 separately for CS+ and CS− following the procedure of Andreatta et al. [38]. Thus, by disentangling the effects, we first found a positive correlation between age and the differential score after Acquisition 1 (*r* (131) = 0.38, *p* < .001), but not after Acquisition 2 (*r* (131) = 0.001, *p* = .987), meaning that the older the participants were, the better they differentiated between the stimuli after Acquisition 1 (Fig. 1). This indicates that discriminative learning went faster in older participants. Second, we found a significant positive correlation with age for CS− ratings (*r* (131) = 0.37, *p* < .001) indicating that the older the participant were, the higher the differences in CS− ratings between Acquisition 2 and Acquisition 1. This indicates that the age differences were due to faster realizing the safety stimulus as safe.

### Generalization phase

#### Ratings

For the arousal, valence, and US expectancy ratings, main effects of stimulus type were found, indicating a downtrend (uptrend, respectively, for valence) from CS+ to CS− (arousal: *F* (4,446) = 34.30, *p* < .001, *η*^2^ = .21; valence: *F* (3,397) = 31.72, *p* < .001, *η*^2^ = .19; US expectancy: *F* (3,350) = 91.35, *p* < .001, *η*^2^ = .41).

Analysis revealed significant linear (arousal: *F* (1,132) = 71.47, *p* < .001, *η*^2^ = .35; valence: *F* (1,132) = 57.46, *p* < .001, *η*^2^ = .30; US expectancy: *F* (1,132) = 153.28, *p* < .001, *η*^2^ = .54) and quadratic trends (arousal: *F* (1,132) = 11.83, *p* = .001, *η*^2^ = .08; valence: *F* (1,132) = 16.26, *p* < .001, *η*^2^ = .11; US expectancy: *F* (1,132) = 47.17, *p* < .001, *η*^2^ = .26) for all ratings.

Participants rated the CS+ as more arousing (*t* (132) = − 8.00, *p* < .001) and more negative (*t* (132) = 7.25, *p* < .001), and showed higher US expectancy to CS+ (*t* (132) = − 12.14, *p* < .001) compared to CS− (Suppl Fig. 3). Moreover, the participants generalized conditioned anxiety to GS1 and GS2, because these stimuli were rated more arousing (GS1: *t* (132) = − 6.05, *p* < .001; GS2: *t* (132) = − 3.57, *p* = .001) and more negative (GS1: *t* (132) = 5.56, *p* < .001; GS2: *t* (132) = 3.00, *p* = .003) and the participants showed higher US expectancy ratings (GS1: *t* (132) = − 9.45, *p* < .001; GS2: *t* (132) = − 4.68, *p* < .001) as compared to CS− (Suppl Fig. 3). No significant differences were found between CS− and GS3, GS4 (all *p* values ≥ .037).

Additionally, a significant main effect of phase (*F* (1,132) = 5.78, *p* = .018, *η*^*2*^ = .04) and a significant stimulus type × phase interaction effect (*F* (4,540) = 3.89, *p* = .004, *η*^*2*^ = .03) were obtained for the US expectancy ratings. Post hoc tests revealed that the differentiation between stimuli was better after Generalization phase 2 as compared to Generalization phase 1, demonstrated by lower ratings to GSs and CS−, but higher ratings to CS+ (CS+ :*t* (132) = − 1.88, *p* = .062; GS1: *t* (132) = 0.88, *p* = .383); GS2: *t* (132) = 1.73, *p* = .086; GS3: *t* (132) = 3.06, *p* = .003; GS4: *t* (132) = 2.68, *p* = .008, CS−: *t* (132) = 2.53, *p* = .013; Suppl Table 3).

#### Physiological response

The ANOVA on the SCR amplitudes revealed significant main effects of stimulus type (*F* (3,442) = 6.23, *p* < .001, *η*^*2*^ = .05) and phase (*F* (1, 132) = 9.07, *p* = .003, *η*^*2*^ = .06), but not their interaction (*F* (5,606) = 1.05, *p* = .383, *η*^*2*^ = .01).

In line with the ratings, linear (*F* (1,132) = 11.58, *p* = .001, *η*^*2*^ = .08) and quadratic trends (*F* (1,132) = 9.35, *p* = .003, *η*^*2*^ = .07) turned out to be significant. Significant larger SCR was found to the CS+ as compared to CS− (*t* (132) = 2.70, *p* = .008), but no other contrasts turned out significant (all *p *values ≥ .066).

#### Effects of age

Significant main effects of age were found for the arousal ratings (*F* (1,131) = 11.98, *p* = .001, *η*^*2*^ = .08), the valence ratings (*F* (1,131) = 5.93, *p* = .016, *η*^*2*^ = .04), the US expectancy ratings (*F* (1,131) = 15.81, *p* < .001, *η*^*2*^ = .11), as well as for the SCR amplitudes (*F* (1,131) = 18.11, *p* < .001, *η*^*2*^ = .12), but no interaction effects with stimulus type (all *p* values ≥ .424).

The main effects of age on the arousal and the US expectancy ratings as well as the SCR amplitudes reflected negative correlations (arousal: *r* (131) = − 0.29, *p* = .001; US expectancy: *r* (131) = − 0.33, *p* < .001; SCR: *r* (131) = − 0.35, *p* < .001), and the main effect of age on the valence ratings was evident in a positive correlation (*r* (131) = 0.21, *p* = .016), respectively.

**Fig. 2:**
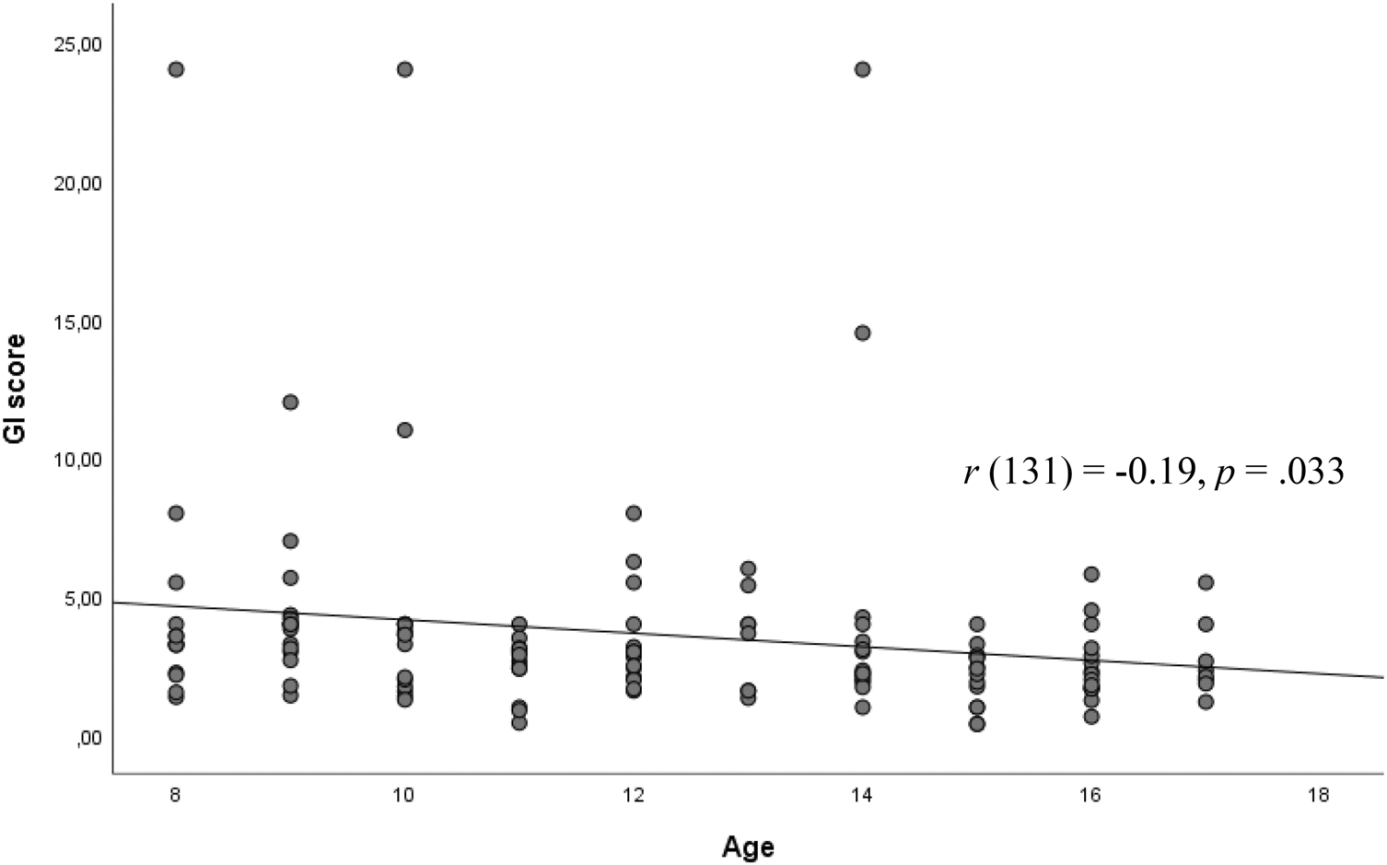
Correlations between age and the generalization index (GI) score based on the US expectancy ratings. The negative correlation indicated that older participants showed reduced generalization of conditioned fear

Following an approach described by Lenaert et al. [39], we then calculated a generalization index (GI) for each participant to provide information about the total amount of generalization for each participant: the sum of the ratings of the GS stimuli (GS1-GS4) was normalized for individual CS+ ratings (GI = [GS1 + GS2 + GS3 + GS4] divided by CS+). Higher GI scores represent stronger generalization. Since there was a significant stimulus × age interaction according to the US expectancy ratings at acquisition, we exemplarily investigated the US expectancy ratings here. We correlated this GS index with age yielding a negative correlation (*r* (131) = − 0.19, *p* = .033, Fig. 2).

## Discussion

Acting on the assumption that the typical onset of anxiety disorders is during childhood [22, 31] and that the overgeneralization of conditioned fear is associated with anxiety disorders in adults [18, 19], the current study investigated fear learning and generalization of conditioned fear in healthy participants, aged 8–17 years. The aim was to examine fear learning and fear generalization in association with age. Therefore, *n* = 133 children and adolescents underwent a discriminative fear conditioning and generalization task measuring the ratings of arousal, valence and US expectancy as well as the electrodermal responses as a physiological index of arousal. In sum, the results demonstrated that the overall pattern of fear learning and generalization was influenced by participants’ age: (1) The ratings of arousal and US expectancy as well as the physiological data (skin conductance response: SCR) were generally lower with increasing age, indicating reduced responses with increasing age to all of the stimuli types, irrespective to their quality. (2) The ratings of US expectancy indicated a better discrimination between CS+ and CS− with increasing age as well as a faster learning, as this correlation was found after Acquisition 1, but not after Acquisition 2. Moreover, (3) the US expectancy ratings indicated reduced overgeneralization with increasing age. The results will be systematically discussed in the following.

Successful acquisition of conditioned fear was indicated by stronger aversive ratings as well as physiological responses for threat vs. safety stimuli, replicating previous studies in children and adolescents using this paradigm [9, 10, 23]. The responses to the generalization stimuli showed a clear generalization for the ratings but no significant generalization effect for the SCR amplitudes. The trend analyses revealed significant quadratic effects for all variables, which is in line with previous findings in healthy adults [19, 23]. The conditioned responses as reflected in the explicit ratings demonstrated that GS1 and GS2, which shared the most properties with the threat stimulus CS+ , were more anxiogenic than CS−, whereas GS3 and GS4, which shared more properties with CS−, were rated more similar to the safety stimulus. Regarding the SCR amplitudes, only the CS+ yielded significantly stronger reactions than the safety stimulus. Since the ratings were collected at the end of each experimental phase, while the physiological responses were measured continuously during the experiment, discrepancies between the physiological responses and the ratings are not uncommon. Thus, discrepancies easily arise between the automatic physiological responses and the cognitive appraisal of the same stimuli [40, 41].

As mentioned above, further analyses revealed that the ratings as well as the physiological responses were modulated by participants’ age. Both the subjective ratings as well as the SCR amplitudes demonstrated main effects of age implying that older participants generally showed smaller fear responses than younger participants. This result is in line with previous studies showing that fear intensity decreases with increasing age [42, 43], and may be interpreted as a developmental reduction in fear-related responses. Concerning the SCR amplitudes, the age effects also fit with the common literature, demonstrating lower responses with increasing age [13, 44]. However, given the cross-sectional nature of our study, it may as well be indicative of a selection effect. Since anxiety disorders usually develop during childhood with a median onset of 13 years [31], the participating adolescents were those, who had not already developed a manifest anxiety disorder. Thus, the adolescents in our sample may have been “healthier” than the participating children, who may still be at larger risk to develop a pathological fear.

Results concerning the US expectancy ratings demonstrated that age modulated the differentiation between the threat and the safety stimuli and the generalization of conditioned fear, respectively. In more detail, according to the US expectancy ratings, older children were better in differentiating between the threat and the safety stimuli. Additionally, adolescents were faster in learning that the CS− predicts safety and the CS+ predicts threat as compared to children. This better and even faster learning process resulted in a lower Generalization Index (GI) score, which indicated that the generalization of conditioned fear decreases with increasing age. In line with Lonsdorf et al. [45], we suppose that the US expectancy ratings reflect a more cognitive understanding than the fear responses, whereas the ratings of arousal and valence indicate more subjective feelings. Arousal represents the fear intensity, whereas valence refers to the quality of the responses. Negative valence would occur, e.g., when the stimulation is aversive (e.g., after threat presentation), whereas positive valence should occur after an appetitive stimulation (e.g., after safety signals). Thus, the US expectancy ratings represent the cognitive development in terms of contingency awareness more than the arousal and valence ratings.

Although the described age effect during generalization was only significant for the US expectancy ratings, possibly due to low statistical power and the large age range, it fits with studies in animals also showing age effects on the generalization profiles [8, 27] and a previous study of our research group demonstrating differences in the generalization of conditioned fear between children and adults [23]. Thus, both human and animal studies suggest that developmental progress reduces the generalization and sharpens the discrimination. As mentioned before, we assume that this is due to maturational effects concerning the prefrontal cortex, which is important for inhibiting fear reactions to stimuli never associated with threat. Hence, future research is needed to address the neurobiological circuits mediating generalization of conditioned fear longitudinally across the life span from childhood over adolescence to adulthood.

Nevertheless, the present study adds to the existing literature on fear learning and generalization in children and adolescence. First, it provides evidence how fear generalization gradients correlate with age in a wide relevant age range during critical periods related to the development of anxiety disorders. Second, the current study replicated findings of previous fear generalization studies in a relatively big cohort of children and adolescence using multiple self-report ratings as well as physiological data and a range of four generalization stimuli. However, as the mechanisms involved in the development of anxiety disorders are complex and rely on the interplay of many variables, much more research is needed, especially, longitudinal and catamnestic follow-up studies.

Thus, although there are some key strengths, several further limitations of the current study deserve discussion, and highlight areas for future research. First, due to the focus on the associations with age, we disregarded the fact that even the “healthy” participants could be more or less anxious in a dimensional manner. This leaves the possibility out of consideration that even supposedly healthy participants could have high anxiety scores when measured by, e.g., the State-Trait Anxiety Inventory for Children [46]. Thus, a logical next step is to analyze fear learning and generalization in a clinical sample of children and adolescence to assess whether fear learning and generalization as measured with the current paradigm differed according to different levels of anxiety. Second, the impact of gonodal hormones as well as hormonal contraceptives was disregarded here. However, this should be considered in further studies, because such hormonal changes could influence fear learning [47, 48]. Third, of note is the fact, that our study has a cross-sectional design. Thus, the question still remains open, if quantitative /qualitative differences during aversive conditioning and generalization found in relation with age as well as in patients with anxiety disorder compared to healthy controls are really a risk marker for anxiety disorder or vice versa. Therefore, longitudinal follow-up studies are required to answer this still open question, which is highly important, especially with respect to preventive and therapeutic approaches. Since there are therapeutic interventions accessible to date, but a considerable number of children do not improve, new markers could lead the way to a “personalized medicine approach” [49, 50].

In sum, we demonstrated that fear learning and generalization is associated with age in underage participants. Especially, contingency awareness in form of the US expectancy ratings demonstrated that age is associated with faster fear learning, better stimulus discrimination, as well as reduced stimulus overgeneralization. We suppose that these age-related results are due to maturational aspects concerning the prefrontal cortex. The results fit with animal research [8, 27] as well as our previous publication [23] demonstrating overgeneralization in children compared to adults. However, neurobiological studies are required to directly relate aspects of brain maturation to the generalization of conditioned fear. Moreover, longitudinal follow-up studies are required to answer the still open question, if overgeneralization of fear constitutes a risk factor for the development of anxiety disorders or whether other aspects of increased vulnerability also modulate the fear generalization processes. This issue is highly important with respect to mechanistic insights, e.g., the contribution of attentional processes or cognitive appraisal [51, 52], but also concerning preventive and therapeutic approaches.

## Supplementary Information

Below is the link to the electronic supplementary material.

Supplementary file1 (DOCX 1138 KB)
