# Supplementary material for: Fear conditioning and stimulus generalization in association with age in children and adolescents
Source: Eur Child Adolesc Psychiatry. 2021 May 13;31(10):1581–90. doi: 10.1007/s00787-021-01797-4 (PMC9532335; doi:10.1007/s00787-021-01797-4)
Supplement: Supplementary file 1 — Supplementary file1 (DOCX 1138 KB) [file 787_2021_1797_MOESM1_ESM.docx]

Supplementary Material

**Table 1.** **Descriptive statistics on the age and sex distribution of the final sample**

| age | male | female | N |
| --- | --- | --- | --- |
| 8 | 9 | 4 | 13 |
| 9 | 8 | 8 | 16 |
| 10 | 7 | 8 | 15 |
| 11 | 7 | 7 | 14 |
| 12 | 9 | 6 | 15 |
| 13 | 5 | 3 | 8 |
| 14 | 4 | 11 | 15 |
| 15 | 5 | 9 | 14 |
| 16 | 5 | 10 | 15 |
| 17 | 4 | 4 | 8 |

*Note*: No significant differences in age groups concerning sex (*χ² (9) = 8.82, p = .454, φ = 0.26*).

**Table 2. Results of ANOVAs at pre- acquisition/acquisition.** Main effects of stimulus type and phase as well as the their interaction effects on arousal, valence and US expectancy ratings as well as on skin conductance response (SCR)

|  | **Stimulus Type** | **Phase** | **Stimulus Type x Phase** |
| --- | --- | --- | --- |
| **Arousal** | *F* (1,32) = 62.03,  *p* < .001, *η*^2^ = .32 | *F* (2,233) = 36.57,  *p* < .001, *η*^2^ = .22 | *F* (2,245) = 24.89,  *p* < .001, *η*^2^ = .16 |
| **Valence** | *F* (1,132) = 20.57,  *p* < .001, *η*^2^ = .14 | *F* (2,232) = 1.65  *p* = .198, *η*^2^ = .01 | *F* (2,264) = 20.23,  *p* < .001, *η*^2^ = .13 |
| **US Expectancy** | *F* (1,132) = 36.09,  *p* < .001, *η*^2^ = .22 | *F* (2,220) = 8.21,  *p* = .001, *η*^2^ = .06 | *F* (2,241) = 39.10,  *p* < .001, *η*^2^ = .23 |
| **SCR** | *F* (1,132) = 5.89,  *p* = .017, *η*^2^ = .04 | *F* (2,237) = 5.21,  *p* = .008, *η*^2^ = .04 | *F* (2,240) = 1.76,  *p* = .178, *η*^2^ = .01 |

**Table 3.** **Means and standard deviations of** **US expectancy ratings after each generalization phase** (ranging from 1 “certainly not/no association“ to 11 “very certain”)

|  | **Generalization 1** | **Generalization 2** |
| --- | --- | --- |
| **CS+ (SD)** | 6.60 (3.07) | 7.08 (3.40) |
| **GS1 (SD)** | 6.05 (3.13) | 5.82 (3.23) |
| **GS2 (SD)** | 4.50 (2.82) | 3.99 (2.85) |
| **GS3 (SD)** | 3.96 (2.59) | 3.27 (2.49)^*^ |
| **GS4 (SD)** | 3.55 (2.28) | 3.02 (2.40)^*^ |
| **CS- (SD)** | 3.54 (2.66) | - 1. (2.40)^*^ |

*Note*: *indicate significant differences between Generalization 1 and 2

**Fig1**

**Fig1.** Schematic overview of the fear conditioning and generalization paradigm. During Pre-Acquisition phase four CS+/CS- were presented. During Acquisition phases 12 CS+/CS- were presented. During Generalization phases 12 CS+/CS- as well as 12 GS1/GS2/GS3/GS4, resulting in 72 stimuli during generalization phases.

**Fig2**

***

***

***

***

*

***

**Fig2.** Average ratings (with standard errors) of arousal, valence and US expectancy and normalized skin conductance responses (SCR) to CS+ (dark bars) and CS- (light bars) after pre-acquisition (Pre), Acquisition 1 (ACQ1) and Acquisition 2 (ACQ2). Discriminative responses were evident after the acquisition phases for ratings and psychophysiological responses. *** *p*<.001; **p*<.05

**Fig3**

**

***

***

***

***

***

***

***

***

***

**Fig3.** Mean ratings (with standard errors) averaged between the two generalization phases for arousal, valence and US expectancy as well as normalized skin conductance responses (SCR) to CS+, GS1-4 and CS-. Generalization of conditioned anxiety was mainly evident on the verbal level. *** *p*<.001; ** *p*<.01
